# Towards evidence‐based integration of services for HIV, non‐communicable diseases and substance use: insights from modelling

**DOI:** 10.1002/jia2.25525

**Published:** 2020-06-19

**Authors:** David W Dowdy, Kimberly A Powers, Timothy B Hallett

**Affiliations:** ^1^ Department of Epidemiology Johns Hopkins Bloomberg School of Public Health Baltimore MD USA; ^2^ Department of Epidemiology UNC Gillings School of Global Public Health Chapel Hill NC USA; ^3^ MRC Centre for Global Infectious Disease Analysis Department of Infectious Disease Epidemiology Imperial College London London United Kingdom

**Keywords:** human immunodeficiency virus, integrated care, health systems, mathematical models

The year 2020 is the designated date for achieving the Joint United Nations Programme on HIV/AIDS 90‐90‐90 targets for human immunodeficiency virus (HIV) diagnosis, treatment and viral suppression [1]; it also marks completion of one‐third of the time allotted (from 2015 to 2030) for achieving the Sustainable Development Goals and the corresponding end of acquired immune deficiency syndrome (AIDS) [2]. Yet the HIV epidemic is far from ended: nearly two million people still acquire HIV infection every year, the number of people living with HIV (PLHIV) continues to increase and new infections are still on the rise in many populations [3]. To date, the response to HIV has largely been an “exceptional” one, with dedicated funders (most notably the President’s Emergency Plan for AIDS Relief) tending to build new structures rather than strengthening the underlying health systems [4]. By some measures, this approach has been exceedingly successful, resulting in over 21 million people receiving antiretroviral therapy (ART) and a corresponding reduction in AIDS mortality [3]. But it is also an approach that may require modification in the coming decade, with progress towards Sustainable Development Goals underway and a concomitant focus on Universal Health Coverage (UHC) emerging [5].

Given the ambitious joint goals of ending AIDS while also achieving good health and wellbeing for all people, it may be instructive to consider the population‐level epidemiologic and economic consequences of the different ways in which services for HIV and other conditions can be integrated, in the context of broader health systems [6]. This Supplement presents a set of articles that explore the potential role of mathematical modelling to address this need.

These articles help illustrate that the concept of “integrated HIV services” itself is not – and need not be – uniform across all situations. For example in settings with generalized HIV epidemics, non‐communicable diseases (NCDs), such as cardiovascular disease (CVD) and cancer, are exacting an increasing toll of morbidity and mortality as populations living with HIV age. As such, implementing routine (or even expanded) diagnostic testing and screening for some of these conditions among PLHIV could be an important step forward in certain settings [7]. In contrast, in settings where HIV is concentrated among people who inject drugs (PWID) and thus overlaps strongly with hepatitis C and risk of drug overdose, integration of HIV services with substance use services and hepatitis C treatment programmes might be the overriding priority [8]. In some settings, services for certain other conditions may be well established, such that integrated care might consist primarily of forming linkages between these services and those for HIV, enabling PLHIV to “link out” and thus access more comprehensive care. In other settings, however, services for other conditions may be more rudimentary, and an important dimension of integration could be in the utilization of HIV facilities to strengthen care for PLHIV while also providing some amount of care for HIV‐negative persons. Regardless of how “integrated HIV services” are conceptualized, integration has the potential to effect synergistic benefits by achieving economies of scope, using the same infrastructure to provide multiple services. Because of this potential benefit, integration of HIV and other services merits careful evaluation.

The articles in this Supplement examine a specific set of issues and perspectives around integration of services for HIV and other conditions. In particular, these articles focus on (1) integration of HIV care with services for NCDs, especially CVD, in settings with a high “dual burden” of HIV and CVD, and (2) integration of HIV and substance use services in populations that can benefit from HIV prevention and treatment as a package that also includes services for substance abuse. Although each individual article addresses a narrowly defined topic, these articles collectively provide important insight into some of the potential epidemiological and economic consequences of moving towards more integrated HIV services. They also illustrate that the landscape of integrating HIV services into broader health systems – and integrating broader healthcare services into HIV‐specific systems – is one that is only beginning to take shape; the need for additional data and corresponding analysis to inform specific policy decisions is urgent.

## INTEGRATION OF HIV AND NCD/CVD CARE IN HIGH‐BURDEN SETTINGS

Kibachio *et al*. [9] use the example of HIV/NCD care in Kenya to highlight some of the key considerations that must be taken into account when modelling the integration of HIV and other services. These authors demonstrate how models can provide support throughout the policy‐making process – from estimating disease burden to elucidating policy options to forecasting comparative epidemiological impact, cost‐effectiveness and budget impact of different potential decisions. Similarly, Kintu and colleagues [10] discuss opportunities, challenges and trade‐offs of integrating NCD and HIV services in sub‐Saharan Africa from a policy perspective – including potential increases in efficiency from leveraging HIV platforms to address NCD management, reductions in quality due to overburdened healthcare staff, potential inequalities given the large burden of NCDs in the general population and the need for additional funding to support integration of services. While highlighting potential pitfalls, both papers hypothesize that the benefits of integration may often outweigh the risks in high‐burden settings – and they provide a roadmap for how quantitative models and innovative policy making can support the process of examining these trade‐offs.

This hypothesis of a favourable risk‐benefit balance is tested in three modelling papers that seek to determine if adding CVD care to existing HIV services would be an impactful and/or cost‐effective use of resources. Kasaie *et al*. [11] consider screening PLHIV for hypertension and diabetes in the context of outreach campaigns and HIV treatment in the Sustained East Africa Research in Community Health programme in Kenya [12], and Sando *et al*. [13] consider screening persons on ART in Uganda for hypertension, diabetes and high cholesterol and initiating treatment for these conditions where indicated. Both papers find that such programmes may be cost‐effective in circumstances when the costs of CVD treatments are low, effectiveness is high and persons receiving services are otherwise at elevated risk of suffering ill effects of CVD.

While integrated HIV/NCD programmes may be cost‐effective in some settings, the costs of treatment for PLHIV on ART may be high due to contraindications between common medications for NCDs and ART. A third analysis, by Boettiger *et al*. [14], presents such a counterexample. These authors use data from the TREAT Asia HIV Observational Database to inform a 20‐year simulation of adults receiving ART in Thailand. In this simulated cohort, they estimate that the cost of providing statin therapy to reduce the risk of CVD events would be high compared to its effect. As a result, very large reductions in the cost of those statins would be needed for such an approach to be considered cost‐effective under thresholds that are currently thought to be realistic.

The cost and budget impact of an alternative model – of expanding NCD care for all persons in HIV and acute health clinics more generally (i.e. not in a manner that stems solely from HIV platforms) – is estimated in another paper in this Supplement, by Osetinsky *et al*. [15]. The authors argue that costs of expanding NCD care in western Kenya can be mitigated by growing capacity in existing clinics without NCD services, strengthening referral systems and task shifting between healthcare workers with different levels of training. The costs of expanding NCD care in this study were relatively modest on a per‐visit or per‐facility basis, but a comparison to current conditions is difficult because the health benefit and opportunity costs of this expansion are uncertain. The authors note that a major challenge in the status quo “unintegrated” approach is patients’ out‐of‐pocket expenditure to attend clinic visits, especially for patients who would not otherwise make these trips. As noted by both Osetinsky *et al*. and Kibachio *et al*., this represents an argument in favour of prioritizing NCD management for PLHIV, who unlike the general population must already make frequent clinic visits while on ART.

As a whole, these analyses provide support for the principle of leveraging the HIV care platform to offer more services, but they also point towards the need for specific strategies to be evaluated in practice. Notably, none of these modelling papers tackles the question of equity, in that prioritization of NCD care for PLHIV may disproportionately benefit those who already have better access to care. Nor do they compare these strategies for integrating NCD and HIV services against other major elements in the movement towards UHC, such as providing PLHIV with an evidence‐based Essential Health Package – a package that would make certain essential services universally available while limiting services without sufficient evidence for effectiveness or cost‐effectiveness [16].

## INTEGRATION OF HIV AND SUBSTANCE USE SERVICES

As examples of contexts in which integration of services for key populations can form a potentially synergistic package of comprehensive care, two mathematical modelling studies in this Supplement examine intersections between HIV and substance use in Latin America. Cepeda *et al*. [17] model a range of scenarios in which ART and harm reduction services are scaled up among PWID in Tijuana, Mexico, predicting the impact that concomitant scale‐up could have on the incidence of both HIV and overdose. In contrast, Bórquez *et al*. [18] focus on stimulant use and HIV among men who have sex with men and transgender women in Lima, Peru, exploring the impact of HIV pre‐exposure prophylaxis and harm reduction interventions on HIV incidence, suicide and CVD deaths in this population. Though the specifics of their inquiries differ, both articles conclude – perhaps unsurprisingly – that intervention strategies attending to both HIV and substance use could have substantial beneficial impacts on the comorbid conditions evaluated.

As with all models of complex systems, the models of Cepeda et al and Bórquez *et al*. require numerous input values to parameterize their many moving parts and make quantitative predictions under a range of hypothetical scenarios. Many of these inputs – such as the reduction in sexual HIV transmission afforded by adherent ART use – are relatively well‐established after decades of concentrated study. Others, such as the effectiveness against HIV acquisition of interventions reducing stimulant use, are less certain. Fundamentally, the inclusion of comorbid conditions and corresponding intervention types within HIV transmission modelling frameworks represents a relatively new frontier, requiring structural considerations, modelling assumptions and input values for which the requisite empirical evidence is still nascent.

## FACING THE CHALLENGES AHEAD

The papers in this Supplement illustrate the potential value of modelling to inform policy relating to the integration of services for HIV and other conditions. But they also underscore the tremendous amount of work that still needs to be done in this area. Currently, very few data exist as to the effectiveness and costs of specific, scalable programmes that could effectively integrate HIV services and other health systems. Examples of data that could advance this field include: (a) implementation studies with embedded costing analyses of feasible integration programmes, from screening for diabetes and hypertension among PLHIV in care to integrated management of HIV and substance use for people who drink hazardously or use drugs; (b) pragmatic trials [19] of integrated versus stand‐alone services, using patient‐centred endpoints as outcomes to support the hypothesized causal link between effective integration and improved patient outcomes and (c) economic analyses – including collection of data on such processes as implementation, scale‐up and economies of scale and scope – to test hypotheses about the estimated cost of integrated interventions from the provider perspective. Collection of such data in a range of epidemiological and economic settings could bolster the ability of models to project long‐term impact and assess the cost‐effectiveness of such interventions, thereby informing more effective policy and motivating the next generation of data‐driven modelling.

In constructing such policy‐relevant models, it is important to evaluate specific policies with attention to the underlying epidemiological context and existing health system, rather than expecting that conclusions or principles relevant to one setting will necessarily be generalizable to others. It follows that integrating HIV and other services may not be the best use of resources in some cases. While there is strong global momentum towards integrating health systems and providing UHC, there are likely many cases where integrating care may marginalize at‐risk populations, produce regressive outcomes in terms of equitable sharing of health resources, or result in inefficient use of scarce healthcare resources that could be put to better use in other ways. Using models to investigate these unintended effects can help us more transparently and systematically consider the broader consequences – both positive and negative – of specific integration policies in specific settings.

As highlighted in the Viewpoint by Kupfer *et al*. [20], enhanced capacity in analysis and modelling is an essential step towards collection of relevant data and performance of effective analyses to inform in‐country decisions regarding integration of HIV services with broader health systems. These authors highlight the importance of making analytic tools more broadly available, investing in training centres within low‐ and middle‐income countries, and engaging directly with decision makers when constructing policy‐facing analyses.

Finally, the papers in this Supplement highlight the importance of more precise thinking about integrated HIV services and their effects. “Integrated HIV care” is not a single intervention that can be universally applied; rather, this broad term encompasses a wide array of specific intervention and policy options that must be tailored to the appropriate population and evaluated individually. As data on such specific integrated HIV interventions emerge, models will evolve from the more generic approaches taken today to answering more specific research questions to help inform specific sets of decision makers. To be useful, this next generation of models will need to be more carefully calibrated to data for particular populations, more advanced in their ability to incorporate analyses of uncertainty and generalizability to other settings, and more grounded in empirical data about intervention effects (as those data emerge). They must also be more cognizant of potential secondary effects of HIV integration policy; such effects might include (a) adverse consequences to health systems and/or funding streams that are incapable of handling additional capacity and (b) unintended inequities from providing additional services to those who already have better access to other health services (while also acknowledging the potential efficiencies of doing so). The analyses presented in this Supplement are an important first step in the direction of informing HIV integration policy, but there is much more work to be done – in terms of collecting requisite data on effectiveness and costs of specific interventions as well as developing models that can exploit those data to their maximum utility.

In conclusion, this Supplement helps to define a path towards more evidence‐based decision making in the context of integrating services for HIV and other conditions. It is currently hypothesized by many that such integration will lead to better health outcomes for patients and populations and more efficient use of resources. Coupled with collection of empirical data on the costs and effectiveness of specific interventions, models can help us to understand the contexts in which that hypothesis might be supported and those in which integration of HIV and other health services may not be such a priority. Better data and better models can help to define specific policy options and provide evidence as to which of those options should be advanced, and which should be reconsidered. Models are an important component of an evidence‐based decision‐making process for integrated HIV services, but current models also illustrate the urgent need to strengthen the research enterprise responsible for producing the data on which such models rely. In order to end the AIDS epidemic in the next decade while also achieving UHC, we must prioritize the collection of better data on integrated HIV services and the improvement of models themselves – and we must do so well before 2030 approaches.

## Competing interests

Dr. Dowdy is a co‐author on the manuscript written by Kasaie *et al*. [11]. Dr. Powers has no competing interests to declare. Dr. Hallett was the recipient of a grant from Fogarty International that supported the work of one the papers in this Supplement.

## Authors’ Contributions

All authors served as Guest Editors to the Supplement and conceived the editorial. DWD wrote the first draft of the manuscript. All authors revised the manuscript for intellectual content and approved the final version submitted for publication.

## Abbreviations

AIDS, acquired immune deficiency syndrome; ART, antiretroviral therapy; CVD, cardiovascular disease; HIV, human immunodeficiency virus; NCD, non‐communicable disease; PLHIV, people living with HIV; PWID, people who inject drugs; UHC, universal health coverage.

## References

[1] Joint United Nations . Programme on HIV/AIDS (UNAIDS). 90–90‐90: an ambitious treatment target to help end the AIDS epidemic. Geneva: UNAIDS 2014.

[2] United Nations General Assembly . Transforming our world: the 2030 Agenda for Sustainable Development. A/Res/70/1. Geneva: United Nations 2015.

[3] Joint United Nations Programme on HIV/AIDS (UNAIDS) . Global HIV & AIDS statistics – 2019 fact sheet [cited 2020 Jan 25]. Available from: https://www.unaids.org/sites/default/files/media_asset/UNAIDS_FactSheet_en.pdf

[4] Bekker LG , Alleyne G , Baral S , Cepeda J , Daskalakis D , Dowdy D , et al. Advancing global health and strengthening the HIV response in the era of the Sustainable Development Goals: the International AIDS Society—Lancet Commission. Lancet. 2018;392(10144):312–58. 30032975 10.1016/S0140-6736(18)31070-5 PMC6323648

[5] Jay J , Buse K , Hart M , Wilson D , Marten R , Kellerman S , et al. Building from the HIV response toward universal health coverage. PLoS Med. 2016;13:e1002083. 27529809 10.1371/journal.pmed.1002083 PMC4987004

[6] Vorkoper S , Kupfer LE , Anand N , Patel P , Beecroft B , Tierney WM , et al. Building on the HIV chronic care platform to address noncommunicable diseases in sub‐Saharan Africa: a research agenda. AIDS. 2018;32 Suppl 1:S107. 29952796 10.1097/QAD.0000000000001898 PMC6438180

[7] Patel P , Rose CE , Collins PY , Nuche‐Berenguer B , Sahasrabuddhe VV , Peprah E , et al. Noncommunicable diseases among HIV‐infected persons in low‐income and middle‐income countries: a systematic review and meta‐analysis. AIDS. 2018;32 Suppl 1:S5. 29952786 10.1097/QAD.0000000000001888 PMC6380891

[8] Haldane V , Cervero‐Liceras F , Chuah FL , Ong SE , Murphy G , Sigfrid L , et al. Integrating HIV and substance use services: a systematic review. J Int AIDS Soc. 2017;20(1):21585. 28692211 10.7448/IAS.20.1.21585 PMC5515016

[9] Kibachio J , Mwenda V , Ombiro O , Kamano JH , Perez‐Guzman PN , Mutai KK , et al. Recommendations for the use of mathematical modelling to support decision‐making on integration of non‐communicable diseases into HIV care. J Int AIDS Soc. 2020;23(S1):e25505. 32562338 10.1002/jia2.25505 PMC7305412

[10] Kintu A , Sando D , Okello S , Mutungi G , Guwatudde D , Menzies NA , et al. Integrating care for non‐communicable diseases into routine HIV services: key considerations for policy design in sub‐Saharan Africa. J Int AIDS Soc. 2020;23(S1):e25508. 32562370 10.1002/jia2.25508 PMC7305410

[11] Kasaie P , Weir B , Schnure M , Dun C , Pennington J , Teng Y , et al. Integrated screening and treatment services for HIV, hypertension and diabetes in Kenya: assessing the epidemiological impact and cost‐effectiveness from a national and regional perspective. J Int AIDS Soc. 2020;23(S1):e25499. 32562353 10.1002/jia2.25499 PMC7305418

[12] Petersen M , Balzer L , Kwarsiima D , Sang N , Chamie G , Ayieko J , et al. Association of implementation of a universal testing and treatment intervention with HIV diagnosis, receipt of antiretroviral therapy, and viral suppression in East Africa. JAMA. 2017;317(21):2196–206. 28586888 10.1001/jama.2017.5705 PMC5734234

[13] Sando D , Kintu A , Okello S , Kawungezi P , Guwatudde D , Mutungi G , et al. Cost‐effectiveness analysis of integrating screening and treatment of selected non‐communicable diseases into HIV/AIDS treatment in Uganda. J Int AIDS Soc. 2020;23(S1):e25507. 32562364 10.1002/jia2.25507 PMC7305460

[14] Boettiger DC , Newall AT , Chattranukulchai P , Chaiwarith R , Khusuwan S , Avihignsanon A , et al. Statins for atherosclerotic cardiovascular disease prevention in people living with HIV in Thailand: a cost‐effectiveness analysis. J Int AIDS Soc. 2020;23(S1):e25494. 32562359 10.1002/jia2.25494 PMC7305414

[15] Osetinsky B , Mwangi A , Pastakia S , Wilson‐Barthes M , Kimetto J , Rono K , et al. Layering and scaling‐up chronic non‐communicable disease care on existing HIV care systems and acute care settings in kenya: a cost and budget impact analysis. J Int AIDS Soc. 2020;23(S1):e25496. 32562355 10.1002/jia2.25496 PMC7305417

[16] Jamison DT , Alwan A , Mock CN , Nugent R , Watkins D , Adeyi O , et al. Universal health coverage and intersectoral action for health: key messages from Disease Control Priorities. Lancet. 2018;391(10125):1108–20. 29179954 10.1016/S0140-6736(17)32906-9 PMC5996988

[17] Cepeda JA , Bórquez A , Magana C , Vo A , Rafful C , Rangel G , et al. Modelling integrated antiretroviral treatment and harm reduction services on HIV and overdose among people who inject drugs in Tijuana, Mexico. J Int AIDS Soc. 2020;23(S1):e25493. 32562375 10.1002/jia2.25493 PMC7305416

[18] Bórquez A , Rich K , Farrell M , Degenhardt L , McKetin R , Tran L , et al. Integrating HIV pre‐exposure prophylaxis and harm reduction among men who have sex with men and transgender women to address intersecting harms associated with stimulant use: a modelling study. J Int AIDS Soc. 2020;23(S1):e25495. 32562365 10.1002/jia2.25495 PMC7305413

[19] Ford I , Norrie J . Pragmatic trials. N Engl J Med. 2016;375(5):454–63. 27518663 10.1056/NEJMra1510059

[20] Kupfer LE , Beecroft B , Viboud C , Wang X , Brouwers P . A call to action: strengthening the capacity for data capture and computational modelling of HIV integrated care in low and middle income countries. J Int AIDS Soc. 2020;23(S1):e25475. 32562312 10.1002/jia2.25475 PMC7305411

